# Genome-wide investigation and expression pattern of *PHR* family genes in cotton under low phosphorus stress

**DOI:** 10.7717/peerj.14584

**Published:** 2022-12-15

**Authors:** Yan Zhao, Peiyu Li, Huarui Wang, Jiping Feng, Yuxin Li, Shanshan Wang, Yuanjie Li, Yanyan Guo, Lin Li, Yao Su, Zhengwen Sun

**Affiliations:** State Key Laboratory of North China Crop Improvement and Regulation, Key Laboratory for Crop Germplasm Resources of Hebei, College of Agronomy, Hebei Agricultrual University, Baoding, China, China

**Keywords:** Cotton, PHR gene, Transcription factor, Low phosphorus stress, Expression

## Abstract

Phosphorus starvation response (PHR) protein is an important transcription factor in phosphorus regulatory network, which plays a vital role in regulating the effective utilization of phosphorus. So far, the *PHR* genes have not been systematically investigated in cotton. In the present study, we have identified 22, 23, 41 and 42 *PHR* genes in *G. arboreum*, *G. raimondii*, *G. hirsutum* and *G. barbadense*, respectively. Phylogenetic analysis showed that cotton *PHR* genes were classified into five distinct subfamilies. The gene structure, protein motifs and gene expression were further investigated. The *PHR* genes of *G. hirsutum* from the same subfamily had similar gene structures, all containing Myb_DNA-binding and Myb_CC_LHEQLE conserved domain. The structures of paralogous genes were considerably conserved in exons number and introns length. The cis-element prediction in their promoters showed that genes were not only regulated by light induction, but also were related to auxin, MeJA, abscisic acid-responsive elements, of which might be regulated by miRNA. The expression analysis showed that the *GhPHR* genes were differentially expressed in different tissues under various stresses. Furthermore, *GhPHR6*, *GhPHR11*, *GhPHR18* and *GhPHR38* were significantly changed under low phosphorus stress. The results of this study provide a basis for further cloning and functional verification of genes related to regulatory network of low phosphorus tolerance in cotton.

## Introduction

Phosphorus (P) is a necessary mineral nutrient for plant growth and development, playing an important role in plant cell energy metabolism, enzyme reaction and signal transduction. Plants mainly absorb inorganic phosphorus from soil solution through roots. However, about 95~99% of phosphorus easily complexes with iron, calcium and aluminum in acidic and alkaline soils to produce insoluble phosphorus, which is not conducive to plant absorption and assimilation, becoming primary constraint to global crop production (Péret et al., 2011). For crop production, it is necessary to apply excessive phosphorus fertilizer to supplement the available phosphorus in the soil, but this process will cause serious environmental pollution. Therefore, for sustainable production in phosphorus deficient soil, it is necessary to explore and analyze the molecular basis of high-efficiency utilization of available phosphorus.

To adapt to a low phosphorus medium, plants have evolved a set of complex gene regulation networks, among which the most important members related to phosphate absorption and transport involved *PHR1* (phosphate startup response 1), *IPS1* (induced by phosphate startup 1), *miR399* (microRNA399), *PHO2* (phosphate 2) and *PT* (phosphate transport). In this complex regulatory network, PHR protein is an important transcription factor in plant phosphorus regulatory network, which plays a key role in signal transduction and regulation induced by phosphate starvation. At present, *PHR* genes of *Arabidopsis thaliana*, *Oryza sativa*, *Zea mays*, *Glycine max* and other species have been identified (Bustos et al., 2010; Woo et al., 2012; Lin et al., 2013; Guo et al., 2015; Xue et al., 2017; Xu et al., 2018). *Arabidopsis PHR* transcription factor directly regulates gene expression by binding to the sequence of phosphorus starvation induced gene *P1BS* (GNATATNC), and there is functional redundancy among members (Rubio et al., 2001). *Arabidopsis PHR1* and *PHL1* (PHR1-LIKE) transcription factors play a role in plant response to phosphorus starvation. *PHR1* can bind to the promoters of many phosphorus starvation induced genes. The loss of *AtPHR1* gene function in *Arabidopsis* will reshape membrane lipid metabolism, primary and secondary metabolism and photosynthesis, which will affect the growth rate of *Arabidopsis* root and crown and the accumulation of anthocyanins. *PHR1* deletion mutation will affect the expression of some phosphorus starvation induced genes, resulting in the decrease of glucose, fructose, sucrose and starch contents in the mutants (Bustos et al., 2010; Nilsson et al., 2012; Pant et al., 2015). In rice, SPX family proteins are involved in phosphorus sensing and signaling by inhibiting the transcriptional activity of *OsPHR2* (Lv et al., 2014). Rice contains genes *OsPHR1*, *OsPHR2* and *OsPHR3* with MYB-CC domain. The loss of any gene function will inhibit the elongation of rice root hair, and then affect the effective absorption of phosphate by plants. MiRNAs regulate plant response to low phosphorus by down regulating gene transcription (Zeng et al., 2014). MiR399 and miR827 are involved in the response of plants to low phosphorus stress (Pant et al., 2008; Lin et al., 2010). MiR399-PHO2 and miR827-NLA mediate the ubiquitination and degradation of phosphate transporters PHT1 and PHO1, and participate in the systematic regulation of phosphorus balance (Chiou et al., 2006; Liu et al., 2014).

Cotton is an important cash crop and raw material for textile industry in China, and plays an important role in the national economy (Ma et al., 2021). Phosphorus is one of the three necessary nutrient elements for cotton growth and development. It can promote cotton budding and flowering in the middle growth stage, promote cottonseed maturation, increase boll weight and open boll early in the later growth stage, which directly affects the yield and fiber quality of seed cotton. Under low phosphorus stress, the adaptive change of root morphology is an important biological basis for crops to make efficient use of soil phosphorus. It has been found the total root length, total root surface area, lateral root length and lateral root number increase in varying degrees under low phosphorus stress in wheat, maize and rice (Chiou & Lin, 2011; Guo et al., 2015; Sawers et al., 2017). When low phosphorus stress occurs, different crops will form a set of adaptive mechanisms to deal with stress. However, there are few studies on the response to low phosphorus stress of cotton (Lei et al., 2022).

In order to explore the candidate genes of low phosphorus tolerance in cotton, we identified the members of *PHR* gene family, and analyzed their gene structure, cis-acting elements and expression pattern based on four *Gossypium* genomes including the latest published genomes of allotetraploid cotton. It provides a reference for further revealing the biological function of *PHR* transcription factor under low phosphorus stress in cotton.

## Material and method

### Identification and analysis of cotton *PHR* gene family members

To obtain the members of *PHR* family genes, the amino acid sequences with conserved domain Myb DNA-binding (PF00249) and Myb_CC_LHEQLE (PF14379) of the *PHR* transcription factor family were searched from the four *Gossypium* genomes including *Gossypium arboreum* (CRI, version 1.0), *G. raimondii* (JGI, version 2.0), *G. hirsutum*(HEBAU, version 1.0) and *G. barbadense* (HEBAU, version 1.0) (Paterson et al., 2012; Wang et al., 2012; Li et al., 2014; Ma et al., 2021). The obtained sequences of *PHR* transcription factor were identified using SMART (http://smart.embl-heidelberg.de/) and CDD databases (https://www.ncbi.nlm.nih.gov/Structure/cdd/wrpsb.cgi). The physical and chemical properties of the proteins were predicted using the online website ExPASY (https://web.expasy.org/protparam/).

### Conserved domain and gene structure analysis of cotton *PHR* family members

The online website MEME (http://meme-suite.org/tools/meme) was used to predict the conserved motif of cotton *PHR* genes, and the number of motifs was set to 10, other parameters are the default settings. The genes structure of cotton *PHR* transcription factors were draw using online website GSDS (http://gsds.gao-lab.org/) based on cotton genome annotations.

### Phylogenetic relationship of cotton *PHR* gene family members

The protein sequences of *Arabidopsis thaliana PHR* family gene were downloaded from Phytozome database (https://phytozome-next.jgi.doe.gov). The protein sequences of PHR family genes in cotton and *Arabidopsis* were compared by MEGA 11.0 software (Koichiro, Glen & Sudhir, 2021), and the phylogenetic tree was constructed by adjacency method (NJ). The bootstrap value was 1,000, the model is Poisson model. Finally, we showed the results using the online tool iTOL (Letunic & Bork, 2021).

### Analysis of cis-acting elements of promoters of *GhPHR* gene family members

To understand the possible regulation and response mechanism of cotton PHR genes, the 1.5 kb upstream of genome sequences were obtained from each *GhPHRs*, and submitted to PlantCARE website (http://bioinformatics.psb.ugent.be/webtools/plantcare/html/) to predict cis-acting elements of promoters, and finally visualized by TBtools software (Chen et al., 2020).

### Prediction of miRNA-target *GhPHR* genes

According to the principle of sequence complementarity, the regulatory miRNAs of *GhPHR* genes were predicted. The *GhPHR* target genes of miRNA were predicted using psRNATarget online software (https://www.zhaolab.org/psRNATarget/).

### Differential expression analysis of *GhPHR* family members in roots under low phosphorus stress

Tissue expression specificity of *GhPHR* genes were analyzed by using RNA sequencing data of *G. hirsutum* ‘TM-1’ (Zhang et al., 2015) during different development stages and stress treatments downloaded from the NCBI Sequence Read Archive (PRJNA490626). To further screen *GhPHR* genes in response to low phosphorus stress, we generated and analyzed the genes expression in roots based on transcriptome data of a P-resistant accession under P deficient hydroponic conditions. The cotton seeds were grown in germination boxes containing quartz sand, and the seedlings were moved into half-strength Hoagland normal nutrient solution when the cotyledons had fully expanded. After 3 days, the nutrient solution was replaced with P-deficient and P-replete Hoagland nutrient solution, respectively. The roots of three cotton seedings were sampled at 0 day and 15 days for RNA-seq. The gene expression patten was drawn by Hemi 1.0 software (Deng et al., 2014) with log2 (1 + FPKM) values after averaging three replicates. The expression of six *GhPHR* genes were selected for further verification by qRT-PCR. All reactions were performed using the Roche LightCycler96 RealTime PCR System with three independent biological replicates, each with three technical replicates. The expression of *GhUBQ14* was used as the internal control. Relative gene expression values were calculated using the 2^−ΔCT^ method (Schmittgen & Livak, 2008).

## Results

### Identification of cotton *PHR* family gene members

To investigate the copy number variation of the *PHR* genes during cotton evolution, a comprehensive search was conducted for *PHR* genes across four cottons including *G. arboreum*, *G. raimondii*, *G. hirsutum* and *G. barbadense*. A total of 128 *PHR* gene sequences were detected in the four cotton species, 22, 23, 41, and 42 *PHR* genes were identified, respectively. The detailed information was listed in Tables S1 and S2. The results were further verified through the NCBI-CDD and SMART database. The results showed that the numbers of *PHR* genes in two diploid cotton species were almost similar as were those in two tetraploid cotton species. The number of *PHR* family genes in *G. arboretum* and *G. raimondii* were basically half in *G. hirsutum* and *G. barbadense*, indicating that the *PHR* family was relatively conserved, which conforms to the known evolutionary relationship in different cottons (Paterson et al., 2012).

The names of the *PHR* genes were determined according to the gene information of *Arabidopsis* and the locations on the chromosomes. The encoded protein of the *PHR* family genes in *G. hirsutum* contains 236~494 amino acid residues. The relative molecular mass is between 26.53 and 54.30 kDa, and the theoretical isoelectric point is between 5.50 and 9.82. Each of the family members contains Myb DNA-binding and Myb_CC_LHEQLE domain. Fourty-one *PHR* genes of *G. hirsutum* were distributed on 21 chromosomes except A05, A07, D01, D03 and D07 (Table 1). Subgenome A and subgenome D contained 22 and 19 sequences, respectively. The genes number in subgenome A was consistent with the genes number of *GaPHR*, and four *GhPHR* genes from subgenome D were missing compared to *GrPHRs*. This result indicated that subgenome D of *G. hirsutum* might have lost genes due to redundant gene functions during evolution.

**Table 1:** Information of *PHR* gene family in *G. hirsutum*.

A subgenome of *G. hirsutum*					D subgenome of *G. hirsutum*				
Gene name	Gene ID	Protein length	MV (Da)	pI	Gene name	Gene ID	Protein length	MV (Da)	pI
*GhPHR1*	*GhM_A01G2399*	380	42,304.2	7.76	*GhPHR23*	*GhM_D02G1490*	364	40,435.4	6.85
*GhPHR2*	*GhM_A02G0277*	303	33,075.2	6.52	*GhPHR24*	*GhM_D04G2388*	346	38,667.5	9.37
*GhPHR3*	*GhM_A03G1365*	364	40,578.5	6.85	*GhPHR25*	*GhM_D05G1302*	494	54,301.4	6.23
*GhPHR4*	*GhM_A04G1918*	346	38,712.5	9.34	*GhPHR26*	*GhM_D06G2662*	298	32,958.0	5.88
*GhPHR5*	*GhM_A06G2674*	298	32,865.9	6.11	*GhPHR27*	*GhM_D06G2663*	333	35,805.0	8.65
*GhPHR6*	*GhM_A06G2675*	333	35,829.1	8.65	*GhPHR28*	*GhM_D08G0228*	411	46,409.8	7.05
*GhPHR7*	*GhM_A08G0240*	417	47,059.5	6.86	*GhPHR29*	*GhM_D08G1976*	279	31,618.2	9.82
*GhPHR8*	*GhM_A08G2024*	279	31,659.3	9.78	*GhPHR30*	*GhM_D08G2683*	372	41,765.9	8.19
*GhPHR9*	*GhM_A08G2740*	411	46,419.1	8.69	*GhPHR31*	*GhM_D08G2924*	357	39,650.8	8.62
*GhPHR10*	*GhM_A08G2986*	348	38,638.6	8.77	*GhPHR32*	*GhM_D09G1508*	316	36,391.6	8.20
*GhPHR11*	*GhM_A09G1611*	315	36,353.5	8.05	*GhPHR33*	*GhM_D09G1943*	267	30,049.4	8.20
*GhPHR12*	*GhM_A09G2040*	267	29,998.3	8.20	*GhPHR34*	*GhM_D10G0017*	399	44,484.4	5.78
*GhPHR13*	*GhM_A09G2485*	253	27,983.4	5.97	*GhPHR35*	*GhM_D10G1602*	302	33,108.2	6.78
*GhPHR14*	*GhM_A10G0026*	433	48,090.4	5.50	*GhPHR36*	*GhM_D11G1558*	478	52,511.1	5.69
*GhPHR15*	*GhM_A10G1517*	302	33,070.1	6.25	*GhPHR37*	*GhM_D11G3020*	448	49,301.5	6.20
*GhPHR16*	*GhM_A11G1564*	478	52,622.2	5.69	*GhPHR38*	*GhM_D11G3158*	387	43,073.3	7.41
*GhPHR17*	*GhM_A11G3088*	418	46,179.0	5.97	*GhPHR39*	*GhM_D12G2463*	236	26,531.7	8.43
*GhPHR18*	*GhM_A11G3236*	387	43,196.4	6.88	*GhPHR40*	*GhM_D13G0849*	356	39,260.4	8.15
*GhPHR19*	*GhM_A12G2578*	236	26,730.9	8.43	*GhPHR41*	*GhM_D13G1605*	347	38,580.7	8.01
*GhPHR20*	*GhM_A13G0904*	309	34,021.4	8.07					
*GhPHR21*	*GhM_A13G1449*	374	41,288.3	8.17					
*GhPHR22*	*GhM_A13G1727*	347	38,480.6	8.33					

Three *GhPHR* genes were observed on chromosomes A09 and A13, while D09 and D13 only contained two genes. The A01 and A03 chromosomes contained one gene, but no *GhPHR* gene sequence was contained in D01 and D03. These results showed that the *GhPHR* genes might have been lost and duplicated in the process of cotton evolution, indicating a strong correlation between subgenome A and subgenome D (Paterson et al., 2012; Wang et al., 2018).

### Phylogenetic analysis of the *PHR* gene family in cotton

**Figure 1:**
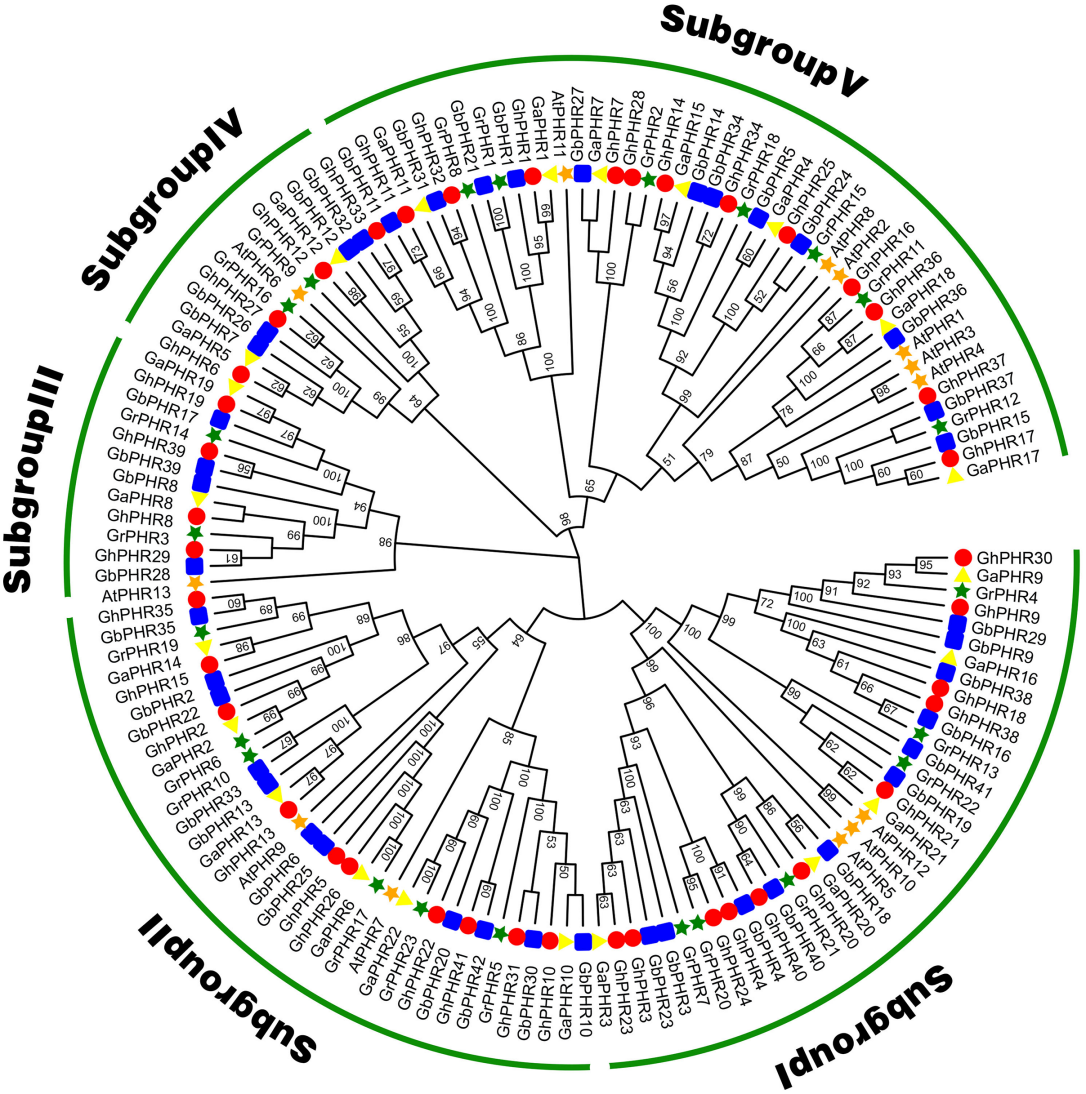
Phylogenic tree of the *PHR* family members in *G. arboreum*, *G. raimondii*, *G. hirsutum*, *G. barbadense* and *Arabidopsis thaliana*. The unrooted phylogenic tree was constructed using MEGA 11.0 by neighbor-joining method. Numbers on branches were bootstrap portions from 1,000 replicates. The subgroups were marked in different colors.

To explore the phylogenetic relationship of the cotton *PHR* genes, we constructed a phylogenetic tree with the neighbor-joining method using 41 *G. hirsutum*, 42 *G. babardence*, 22 *G. arboreum*, 23 *G. raimondii* and 13 *Arabidopsis* PHR amino acid sequences (Fig. 1). All the *PHR* genes can be divided into five subgroups. The *PHR* genes number of *G. hirsutum* and *G. barbadense* was basically twice of *G. arboreum* and *G. raimondii* in each subgroup. This result was consistent with the previous analysis and conformed to the conserved evolutionary relationship in cottons. Among these subgroups, the largest subgroup V consisted of 12 *GhPHRs*, 12 *GbPHRs*, seven *GaPHRs*, seven *GrPHRs* and six *AtPHRs*, showing that has expanded considerably in two allotetraploid cottons. In contrast, subgroup III only included four *GhPHRs*, four *GbPHRs*, two *GaPHRs*, two *GrPHRs* and one *AtPHRs*, indicating might be a highly-conserved clade.

### Analyses of gene structures and protein motifs of *PHR* genes in *G. hirsutum*

**Figure 2:**
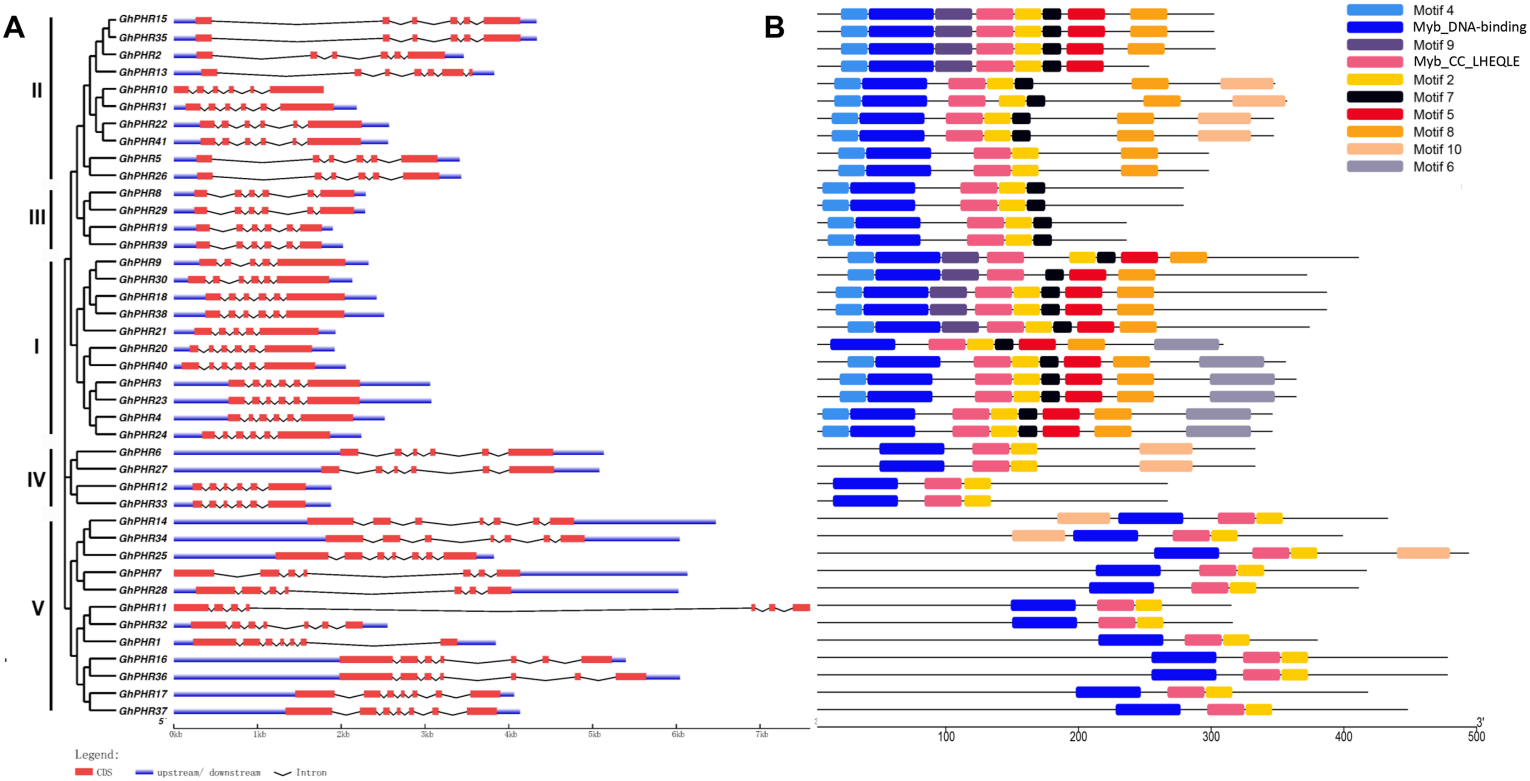
Distributions of gene structure and conserved protein motifs in *GhPHR* genes. (A) Gene structure of all *GhPHR* genes. (B) Conserved protein motifs of all *GhPHR* genes. The red boxes and gray lines represent the exon and intron, respectively. The lengths of the boxes and lines were scaled based on the length of the genes. Conserved motifs in the GhPHR proteins are indicated by colored boxes.

Gene structure analysis of *PHR* gene family members showed that the gene structure of *GhPHR* members in the same subgroup was similar, and there was little difference in the number of exons of *GhPHR* members among different subgroups. Except for the *GhPHR* genes of class V which contained seven exons, most of the other *GhPHR* genes contained six exons (Fig. 2). Furthermore, these PHR protein sequences were submitted to MEME to discover conserved motifs. The adjacent clades carried similar motifs. Analysis of the conserved domains of *GhPHR* gene family members showed that Myb_DNA-binding and Myb_CC_LHEQLE were present in all GhPHR proteins and other motifs were functionally unknown motifs (Fig. 2). Among these, motif 4 occurred in class I, class II and class IV subgroups, motif 6 was unique to class II subgroup, motif 10 were unique to class IV subgroup, and motif 5 was unique to two of class I class II subgroup. It is worth noting that the GhPHR protein motif types of class II were different. Among them, there were eight motifs in *GhPHR2*, *GhPHR15* and *GhPHR35*, only six motifs in *GhPHR5* and *GhPHR26*, and seven motifs in the other five *GhPHRs*. These special conserved motifs may be the main factors enabling *GhPHRs* to participate in different biological functions.

### Analysis of cis-acting elements in the promoter of *GhPHR* family genes

**Figure 3:**
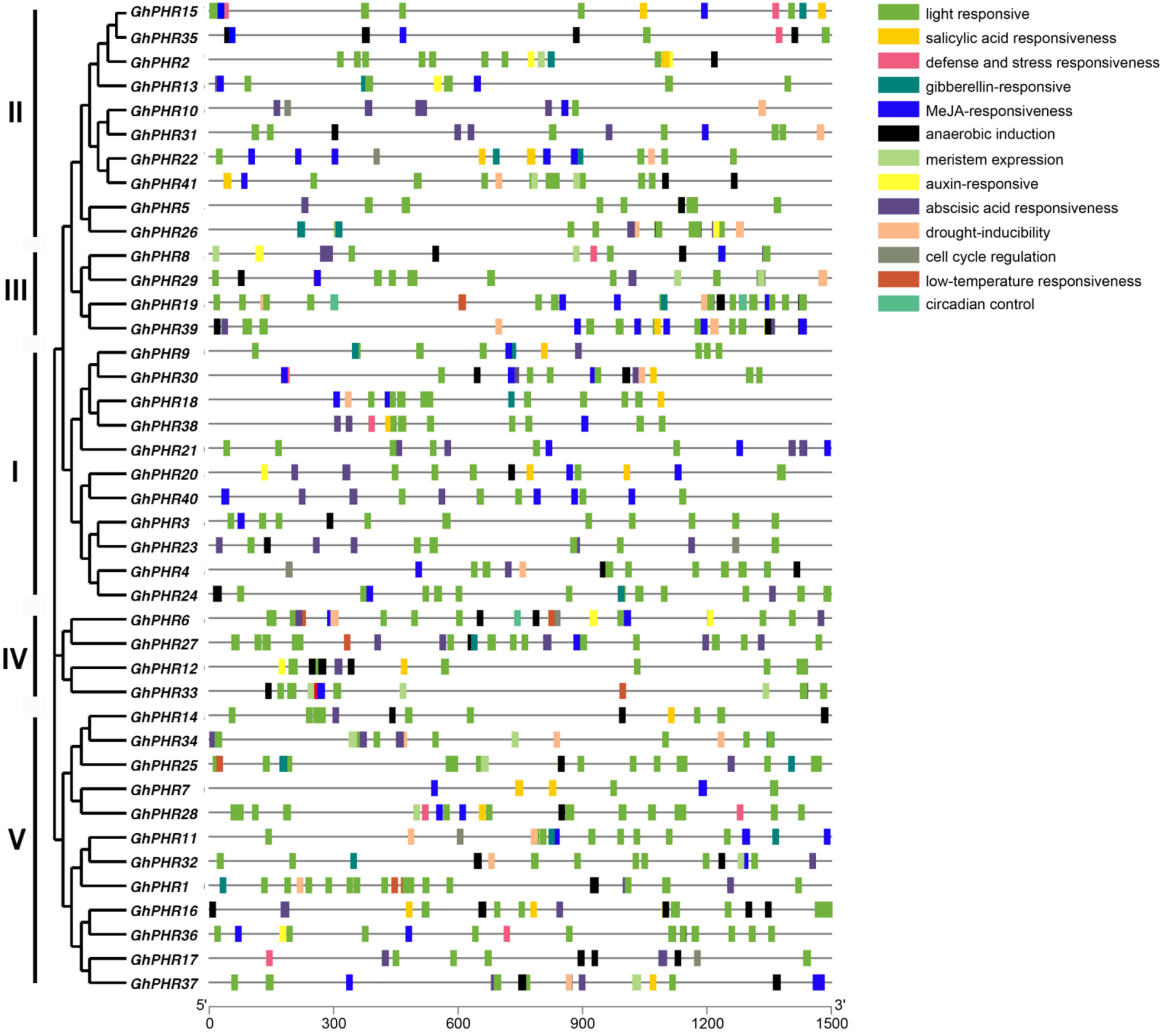
Cis-acting element analysis of *PHR* family members in *G. hirsutum*. The colored boxes indicate different cis-elements in promoters of genes.

To further clarify the possible regulatory mechanism of *GhPHR* family genes under abiotic stress, the promoter sequences were analyzed by using PlantCARE database. The results showed 13 types of cis-acting elements, including light responsiveness, salicylic acid responsiveness, gibberellin-responsiveness, MeJA-responsiveness, anaerobic induction, auxin-responsiveness, abscisic acid responsiveness and so on (Fig. 3). In terms of composition and quantity, the *GhPHR* genes contained an average of 18 cis-acting elements, all of which had light responsiveness elements. Among them, *GhPHR6* and *GhPHR19* contained the most types of response elements (10 kinds), while *GhPHR3*, *GhPHR5*, *GhPHR7* and *GhPHR40* contained the least cis-acting elements (three kinds). In terms of element types, 17 genes contained gibberellin—responsiveness elements including *GhPHR1*, *GhPHR12* and *GhPHR34*. Thirty-one genes contained anaerobic induction elements including *GhPHR5*, *GhPHR19*, *GhPHR24*, and 11 genes contained auxin-responsiveness elements including *GhPHR16*, *GhPHR20*, *GhPHR36*. The results showed that the *GhPHR* genes were not only regulated by light induction, but also played a role during drought, anaerobic and other stresses. Among them, the promoter regions of *GhPHR3*, *GhPHR5*, *GhPHR7* and *GhPHR40* contained less cis elements, which were only related to light, MeJA, abscisic acid and anaerobic induction.

### Prediction of the regulatory miRNA of *PHR* gene family

The online software psRNATarget was used to predict and analyze the regulatory miRNAs of *GhPHR* genes. The regulatory combinations of 33 miRNAs and *PHR* genes were predicted (Table S3). It was found that 12 miRNAs could regulate 16 *PHR* genes. Unpaired energy (UPE) is the energy required to unlock the secondary structure of the target gene miRNA target site. A lower UPE value indicates that miRNA is more likely to bind or cleave the target gene. This study showed some of the predicted results with an expected value less than or equal to 5. *GhPHR21* and *GhPHR32* can be recognized by miR396 and miR7510a, miR2949 and miR7491 at the same time, respectively, which may be regulated by these two miRNAs. *GhPHR19* may be regulated by the sequence cleavage of miR482, *GhPHR4* and *GhPHR24* may be regulated by the transcriptional inhibition of miR827, *GhPHR17* and *GhPHR37* may be regulated by the sequence cleavage of miR2948-5p, and *GhPHR23*, *GhPHR25* and *GhPHR40* may be regulated by the transcriptional inhibition of miR2948-5p. In this study, the regulation modes of interaction combinations were different. Approximately 2/3 of the regulation modes belonged to sequence cleavage and 1/3 belonged to transcriptional inhibition (Table 2).

**Table 2:** Bioinformatic analysis of partial miRNA target sites.

miRNA	Target genes	Expectation		Start	Sequence	End	Inhibition mode
miR827a	*GhPHR4*	5.0	miRNA	1	UUAGAUGACCAUCAACAAACA	21	Translation
					: ::::::: ::::::.:.:		
			Target	952	GGAUUGUUGA-GGUCAUUUGA	971	
miR827b	*GhPHR4*	5.0	miRNA	1	UUAGAUGACCAUCAACAAACA	21	Translation
					: ::::::: ::::::.:.:		
			Target	952	GGAUUGUUGA-GGUCAUUUGA	971	
miR827c	*GhPHR4*	5.0	miRNA	1	UUAGAUGACCAUCAACAAACA	21	Translation
					: ::::::: ::::::.:.:		
			Target	952	GGAUUGUUGA-GGUCAUUUGA	971	
miR7491	*GhPHR11*	4.0	miRNA	1	UGGGAUCUUCGAGAGGAUUGAGCC	24	Translation
					::::::.: :::::::.:		
			Target	324	CCAGAAAUCCUUUGAAAGAUCCUA	347	
miR2949b	*GhPHR12*	4.0	miRNA	1	UCUUUUGAACUGGAUUUGCCGA	22	Translation
					..:.:::: :::::::::		
			Target	497	AGUCUGAGUCCAAUUCAAAAGA	518	
miR2949c	*GhPHR12*	4.0	miRNA	1	UCUUUUGAACUGGAUUUGCCGA	22	Translation
					..:.:::: :::::::::		
			Target	497	AGUCUGAGUCCAAUUCAAAAGA	518	
miR2949a-5p	*GhPHR12*	5.0	miRNA	1	ACUUUUGAACUGGAUUUGCCGA	22	Translation
					..:.:::: ::::::::		
			Target	497	AGUCUGAGUCCAAUUCAAAAGA	518	
miR482a	*GhPHR19*	5.0	miRNA	1	UCUUUCCUACUCCUCCCAUACC	22	Cleavage
					::::: .:::: :::.:::		
			Target	620	AUUAUGGAGGGAGAUGGAGAGA	641	
miR7510a	*GhPHR21*	4.5	miRNA	1	AAGGUCAUGAUCUUUAGCGGCGUU	24	Cleavage
					::.: : ::..::::. ::.::::		
			Target	88	AAUGGCACUGGAGAUUCUGGCCUU	111	
miR396a	*GhPHR21*	5.0	miRNA	1	UUCCACAGCUUUCUUGAACUG	21	Cleavage
					:: ::::::.::. :::::		
			Target	560	AAGCUCAAGAGAGUCUUGGAA	580	
miR396b	*GhPHR21*	5.0	miRNA	1	UUCCACAGCUUUCUUGAACUG	21	Cleavage
					:: ::::::.::. :::::		
			Target	560	AAGCUCAAGAGAGUCUUGGAA	580	
miR2948-5p	*GhPHR40*	4.5	miRNA	1	UGUGGGAGAGUUGGGCAAGAAU	22	Translation
					::: ::::.. :::::..:::		
			Target	46	UUUCCUGCCUGCCUCUCUUACA	67	

### Tissue expression analysis of *GhPHR* family genes

**Figure 4:**
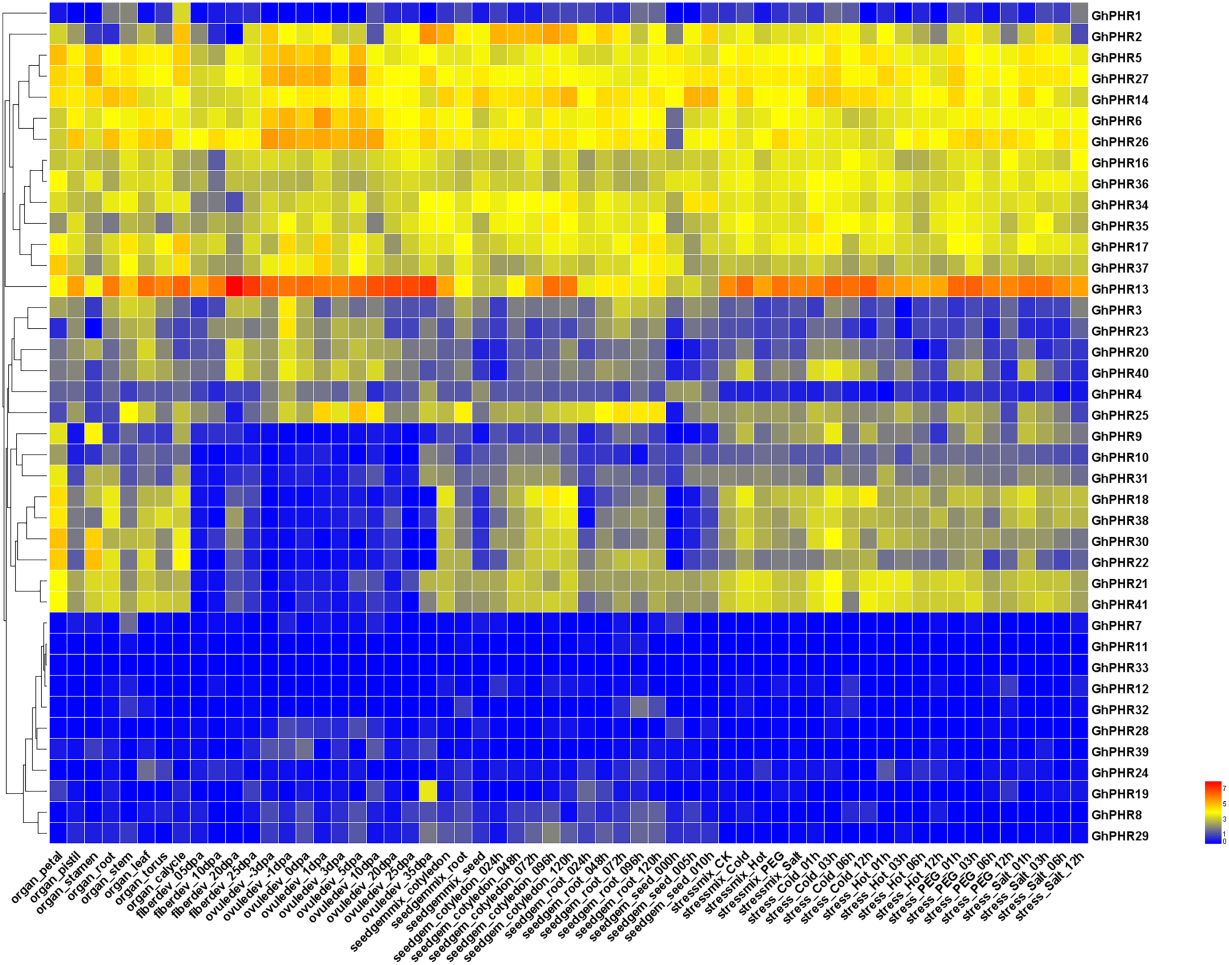
Expression profiles of *GhPHR* genes in different tissues. The color represents the gene expression level based on public transcriptome data. The red and blue colors indicate a high and a low expression level, respectively.

The expression of *GhPHR* family genes was investigated across different tissues and developmental stages of upland cotton. Most of these genes were expressed at varying levels across different tissues and developmental stages (Fig. 4). It was found that five *GhPHR* genes were the most commonly expressed in all tissues. A total of 13 *GhPHRs* were highly expressed in all tissues, indicating that these genes play an important role in all morphogenesis of cotton. Among them, *GhPHR5* and *GhPHR6* were highly expressed in leaves, while *GhPHR13*, *GhPHR14*, *GhPHR26* and *GhPHR27* were highly expressed in both roots and leaves. Combined with the cis-element structure of *GhPHR* promoters, it was speculated that they may play an important role in leaf photosynthesis. A total of eight *GhPHRs* were expressed across different tissues except fiber developmental stages, and the expression of other six *GhPHR* genes were low in different tissues. Altogether, the expression profiles of *GhPHR* genes showed that it plays a role in different tissues of cotton, among which *GhPHR2*, *GhPHR5*, *GhPHR6* and *GhPHR13* have obvious tissue expression specificity.

### Expression pattern of *GhPHR* genes in roots under low phosphorus stress

**Figure 5:**
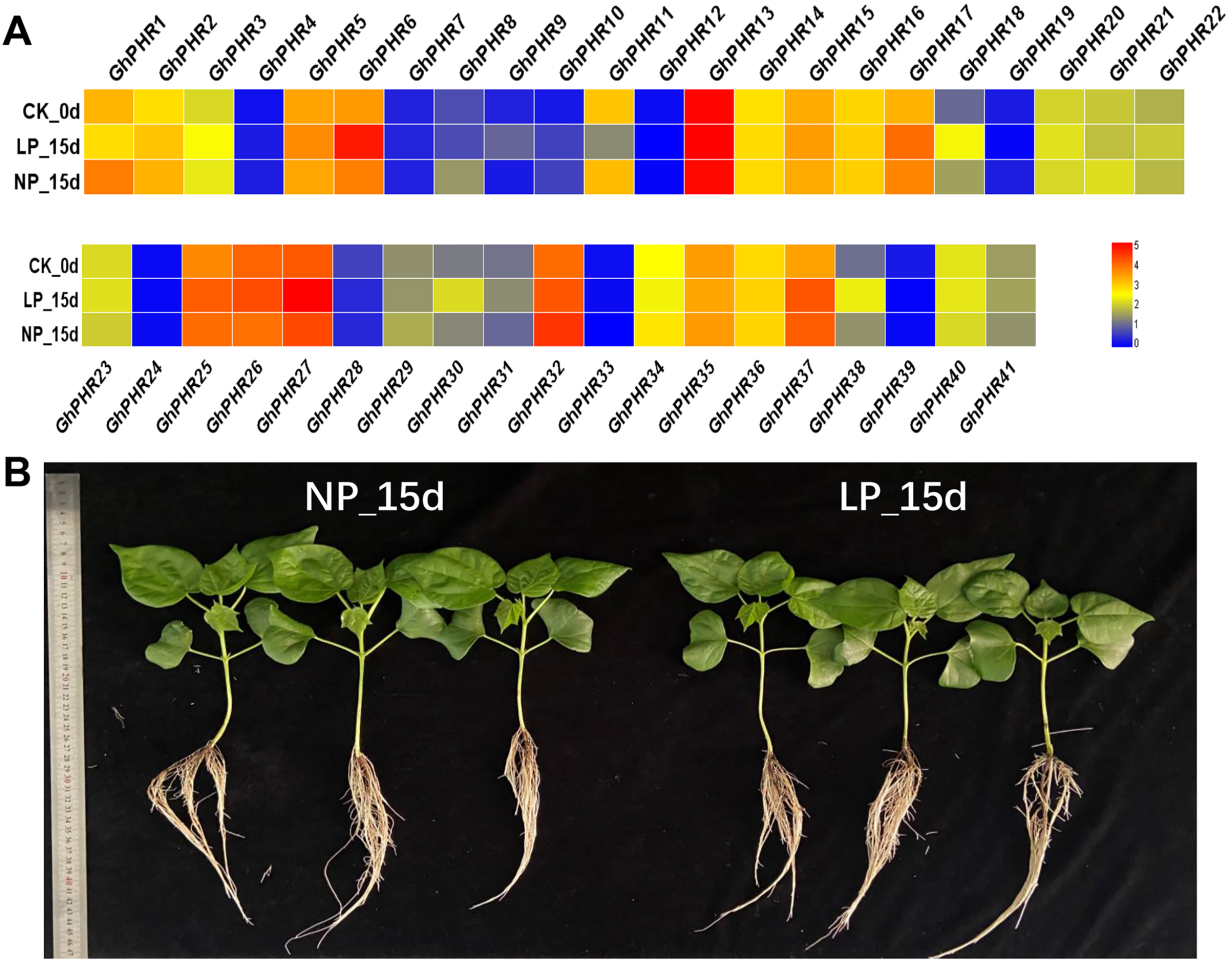
Expression pattern of *GhPHR* genes in root under low phosphorus stress. (A) Expression level of *PHR* genes at 0 d and 15 d under low and normal phosphorus condition, respectively. (B) Cotton seedlings of 15 d under low and normal phosphorus condition. The colorful scale of heat map indicates the relative expression levels where blue indicates low and red indicates high.

**Figure 6:**
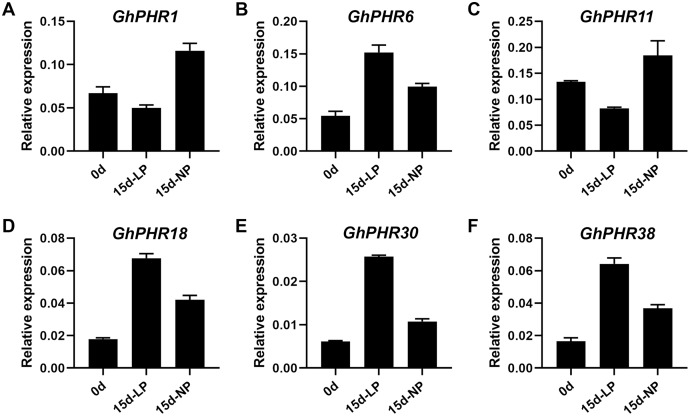
Relative expression level of six representative *GhPHR* genes. (A) *GhM_A01G2399*. (B) *GhM_A06G2675*. (C) *GhM_A09G1611*. (D) *GhM_A11G3236*. (E) *GhM_D08G2683*. (F) *GhM_D11G3158*. Data represent mean ± SE of three biological replicates by qRT-PCR.

The expression of 41 *GhPHR* genes in roots showed that most *GhPHR* genes were affected under low phosphorus treatment, except that *GhPHR4*, *GhPHR7*, *GhPHR12*, *GhPHR19*, *GhPHR24*, *GhPHR33* and *GhPHR39* were not detected (Fig. 5). The expression of *GhPHR1* and *GhPHR11* decreased under low phosphorus stress but that of *GhPHR3*, *GhPHR6*, *GhPHR17*, *GhPHR18*, *GhPHR27*, *GhPHR30* and *GhPHR38* increased. Among them, expression level of *GhPHR17*, *GhPHR30* and *GhPHR38* was significantly higher than that before stress treatment. In addition, *GhPHR5*, *GhPHR13*, *GhPHR15* and *GhPHR26* maintained a high expression level. It should be noted that the expression of *GhPHR11* was significantly lower under low phosphorus stress than under normal phosphorus treatment, and that of *GhPHR18* was significantly higher than that of normal phosphorus treatment (Fig. 5). Further, we selected six differentially expressed *GhPHR* genes for verification by qRT-PCR, showing a consistent trend (Fig. 6). This provided further evidence that the six putative genes were closely associated with low-phosphorus tolerance.

## Discussion

Phosphorus deficiency is a major factor limiting crop yield. In cotton, the *PHR* genes have not been systematically investigated. In the present study, we have identified 22, 23, 41 and 42 *PHR* genes in *G. arboreum*, *G. raimondii*, *G. hirsutum* and *G. barbadense*, respectively. The *GhPHR* genes are differentially expressed in different tissues under various stresses. Furthermore, *GhPHR6*, *GhPHR11*, *GhPHR18* and *GhPHR38* were significantly changed under low phosphorus stress. These results provided a basis for low phosphorus tolerance in cotton.

Plants have evolved a series of morphological, physiological and molecular strategies to adapt to phosphorus deficiency (Veneklaas et al., 2012), including symbiosis with mycorrhizal fungi, secretion of organic acids, remodeling of root structure, and improving the expression of phosphorus transporters (Chiou & Lin, 2011; Sawers et al., 2017). Most of these strategies improve the utilization efficiency of phosphorus by enhancing the mobility of phosphorus in soil or the acquisition of phosphorus by roots. In recent years, genes and proteins related to low phosphorus stress have been found and identified. Among them, *PHR* is a MYB transcription factor, which plays an important role in plant response to low phosphorus stress (Bustos et al., 2010; Sega & Pacak, 2019). It has been reported that *PHR1* and *PHR1-like* genes play a key role in the phosphorus signal regulation network of plants such as *Arabidopsis* (Karthikeyan et al., 2007), rice (Guo et al., 2015), soybeans (Xue et al., 2017), wheat (Chiou & Lin, 2011), maize (Lin et al., 2013; Sawers et al., 2017) and rape (Ren et al., 2012). In addition, genome-wide transcriptional analysis of *Arabidopsis* and rice showed that most phosphorus starvation response genes were induced and activated by *AtPHR1* and *OsPHR2* and their homologous genes *AtPHL1*, *AtPHL2*, *OsPHR1* and *OsPHR3* (Guo et al., 2015; Sun et al., 2016). In our study, the expression of *GhPHR1* and *GhPHR11* decreased but that of *GhPHR6*, *GhPHR18*, *GhPHR30* and *GhPHR38* increased under low phosphorus stress, we further verified the six genes by qRT-PCR, indicating closely associated with low-phosphorus tolerance.

Cis-acting elements regulate gene transcription by responding to different external signals, and then affect plant growth and development (Schmitz, Grotewold & Stam, 2022). Phosphorylation signal transduction and phosphorus starvation response are affected by light, sugar, plant hormones (auxin, ethylene, cytokinin and gibberellin), as well as oxygen (Karthikeyan et al., 2007; Lei et al., 2011; Klecker et al., 2014). For example, the expression of *AtPHR1* is regulated by light and ethylene, and the response to phosphorus starvation is regulated by the promoter of *AtPHR* gene (Liu et al., 2017). In this study, 13 types of cis-acting elements were identified in the promoter of *PHR* genes. A large number of light response elements and hormone elements showed that the expression and regulation of *PHR* genes were affected by light and hormone. MiRNAs regulate plant response to low phosphorus by down regulating gene transcription (Zeng et al., 2014). MiR399 and miR827 are involved in the response of plants to low phosphorus stress (Pant et al., 2008; Lin et al., 2010). In this study, 12 cotton miRNAs such as miR396, miR482 and miR827 have the potential to regulate *GhPHR* genes, which may play a role in phosphorus absorption and transport in cotton.

Most *PHR* genes in maize, rice and sorghum are continuously expressed in all tissues, indicating that they may play an important role in regulating phosphorus uptake and transport (Lin et al., 2013; Xu et al., 2018). This study analyzed the tissue expression of cotton *PHR* family genes in roots, stem, leaves and so on, and found that there was tissue-specific expression of cotton *PHR* family genes, which was similar to that of other crops (Lin et al., 2013). In tissue expression analysis, it was found that the expression of *GhPHRs* in root was high, and there were gibberellin and auxin response elements related to stress resistance in the cis-acting elements of promoter. In addition, the expression of *GhPHRs* exceeded the expression level before stress after low phosphorus stress, so it is speculated that *GhPHRs* may be related to the remodeling of root morphology under abiotic stress.

In conclusion, 128 *PHR* genes were identified in cotton, 41 of which were in *G. hirsutum*. There *GhPHR* genes had great differences in the number of amino acids and isoelectric point characteristics. In addition, the promoter region of *GhPHRs* has different cis-acting elements related to light response, and biotic and abiotic stresses. Further, expression analysis of the genes of showed that *GhPHR11* and *GhPHR18* were significantly highly expressed in root under low phosphorus stress. This study has provided a foundation for subsequent functional study of *PHR* genes and the breeding of new cotton varieties.

## Supplemental Information

10.7717/peerj.14584/supp-1 Supplemental Information 1 PHR genes in the four cotton species.

10.7717/peerj.14584/supp-2 Supplemental Information 2 Raw data for qPCR.

## Additional Information and Declarations

### Data Availability

The following information was supplied regarding data availability: The raw measurements are available in the Supplemental Files.
